# The Efficacy of Music for Emotional Wellbeing During the COVID-19 Lockdown in Spain: An Analysis of Personal and Context-Related Variables

**DOI:** 10.3389/fpsyg.2021.647837

**Published:** 2021-04-09

**Authors:** Pastora Martínez-Castilla, Isabel M. Gutiérrez-Blasco, Daniel H. Spitz, Roni Granot

**Affiliations:** ^1^Department of Developmental and Educational Psychology, Universidad Nacional de Educación a Distancia, Madrid, Spain; ^2^Independent Researcher, Málaga, Spain; ^3^Department of Psychology and The Jerusalem School of Business Administration, The Hebrew University of Jerusalem, Jerusalem, Israel; ^4^Department of Musicology, The Hebrew University of Jerusalem, Jerusalem, Israel

**Keywords:** COVID-19, music, efficacy, emotional wellbeing, affect regulation, Spain

## Abstract

The strict lockdown experienced in Spain during March–June 2020 as a consequence of the COVID-19 crisis has led to strong negative emotions. Music can contribute to enhancing wellbeing, but the extent of this effect may be modulated by both personal and context-related variables. This study aimed to analyze the impact of the two types of variables on the perceived efficacy of musical behaviors to fulfill adults’ emotional wellbeing-related goals during the lockdown established in Spain. Personal variables included age, gender, musical training, personality, resilience, and perception of music’s importance. Contextual variables referred to living in a region with a high COVID-19 impact, perception of belonging to a risk group, being alone, having caring responsibilities during confinement, and amount of time of music listening as compared to prior to the crisis. The study was conducted retrospectively during August–December 2020, when the strict lockdown was over in Spain. An online survey was disseminated among the general population and groups of musicians, and the answers of 507 adults (from 18 years on, 73.9% females, 51.3% musically trained adults) were analyzed. Only personal, but not COVID-19 context-related variables, showed an impact on music’s efficacy. The youngest age group of adults and those with musical training reported the highest efficacy of music for wellbeing enhancement, and music’s importance was found to be the main significant predictor of music’s perceived efficacy. Our findings suggest that the people who have been reported to be emotionally more vulnerable during the lockdown, due to either a strong impact on their daily lives or their lower resilience, perceive a higher benefit from musical behaviors. Being musically trained, even for a small number of years, also leads to a perception of higher efficacy of music for the achievement of emotional wellbeing goals. However, this effect is explained by the musically trained individuals’ higher perception of music’s importance. Although musical behaviors can be generally considered as important for wellbeing enhancement, our study highlights who are the potential individuals who could benefit the most from music-related activities for obtaining better levels of wellbeing, at least within the current context of the COVID-19 crisis.

## Introduction

The COVID-19 pandemic has caused a significant impact on people’s lives. As a measure to control the crisis, lockdowns have been imposed worldwide, which have generally derived into reduced emotional wellbeing (Cénat et al., 2020). Lockdown restrictions, duration, and timing varied across countries, some of them included very strict limitations (Thomas et al., 2020). This was the case of Spain, where an emergency status was declared on 14 March 2020 and a severe quarantine took place until 21 June 2020 (Boletín Oficial del Estado, 2020a, b). Spanish citizens were not allowed to leave their homes unless they were considered essential workers, went out for food shopping or for health issues (Boletín Oficial del Estado, 2020a, b). Similarly to what has been reported in other places (Brailovskaia et al., 2020; Cénat et al., 2020), the negative psychological impact of the lockdown in Spain has already been highlighted (Giménez-Dasí et al., 2020; González-Sanguino et al., 2020; Justo-Alonso et al., 2020; Sandín et al., 2020). Among other emotional symptoms, high levels of depression, anxiety, stress, worries, feelings of loneliness and sleep problems have been reported in adults of various ages (Justo-Alonso et al., 2020; Sandín et al., 2020). Individual differences related to personal and contextual variables have also been noted. Within the former, the negative emotional impact is higher in women than in men, and in younger than in older adults (González-Sanguino et al., 2020; Justo-Alonso et al., 2020; Sandín et al., 2020). Resilience -the capacity of dealing with adversities as challenges- has been found to be a protective factor of the psychopathologies related to the pandemic (Luceño-Moreno et al., 2020). Variables linked to context of the lockdown include media exposure, having a close relative infected, perception of being at risk, caring responsibilities, living alone, or the COVID-19 incidence regional rate; the higher the levels in these variables, the higher the negative impact on emotional wellbeing (Balluerka-Lasa et al., 2020; Fullana et al., 2020; González-Sanguino et al., 2020; Luceño-Moreno et al., 2020; Sandín et al., 2020).

In times when psychological welfare is threatened, research on strategies that can be helpful to promote positive emotions is of great relevance. Among those, music-related activities are considered especially useful, being these available to everyone and an easy and low cost means to enhance wellbeing (Dingle et al., 2017; Daykin et al., 2018; Kappert et al., 2019; de Witte et al., 2020; Sheppard and Broughton, 2020). Not in vein, when considering the psychological functions of music, the socioemotional uses of musical behaviors (e.g., either music listening or music performance) have been traditionally highlighted (Hargreaves and North, 1999; Schäfer et al., 2013). A range of functions are here included, such as affect management, interpersonal relationships, enjoyment and entertainment, anxiety and stress reduction, or diversion (e.g., Toyoshima et al., 2011; Sanal and Gorsev, 2014; Schäfer and Eerola, 2020). The efficacy of musical behavior to fulfill these functions could be accounted for by considering the reciprocal-feedback model of musical response (Hargreaves, 2012; Hargreaves et al., 2016). According to this model, musical response depends on three different components: the music, the person, and the context. Music variables include genre, style, complexity, or prototypicality, among others. The variables referred to the person include those such as gender, age, or musical experience. Context refers to variables linked to culture and social settings, everyday situations, or ongoing activities. The music and person shape preference and taste development, while the person and context modulate the use of musical behavior for the achievement of goals in specific environments (Hargreaves, 2012). Therefore, although music features have an impact on the emotional response (Garrido and Schubert, 2015), the person and context seem to be particularly relevant for the study of the efficacy of musical behavior for emotional wellbeing.

The literature on music listening has been very fruitful in the analysis of the effects of personal and contextual variables (e.g., Greb et al., 2018, 2019), although similar results are found when considering music performance. Personality traits, gender, age, musical training, and perception of music’s importance in life have been studied among the former. Emotional stability predicts the emotional use of music listening (Chamorro-Premuzic et al., 2012). Introversion is also related to this music function (Chamorro-Premuzic and Furnham, 2007; Chamorro-Premuzic et al., 2012), although cross-cultural differences have been reported. For Spanish adults, extraversion, but not introversion, is the trait related to affect regulation through music (Chamorro-Premuzic et al., 2009a). Regarding gender, a body of research has shown that women are more likely than men to listen to music for enjoyment, consolation, releasing negative emotions or reducing loneliness (North, 2010; Lonsdale and North, 2011; Chamorro-Premuzic et al., 2012; Ter Bogt et al., 2017). Similarly, women who sing or play an instrument report higher levels of wellbeing than men (Krause et al., 2019). However, inconsistent results have also been found in this regard. Other studies have shown that the benefits of singing or those of music listening for emotional wellbeing are unaffected by gender (Livesey et al., 2012; Saarikallio, 2012). Studies on the effect of age seem to be complex. Young adults (below 30 years) mainly use music listening for affect regulation and social connection, while older adults often use music for cognitive and eudemonic reasons (e.g., personal growth) (Lonsdale and North, 2011; Groarke and Hogan, 2016). However, other studies have reported that older people can also make an extensive emotional use of music listening (Saarikallio, 2011). Furthermore, when negative affect is induced, music listening leads to a greater reduction of negative affect in older adults as compared to younger ones (Groarke and Hogan, 2019). Younger and older adults similarly profit from singing for wellbeing enhancement (Livesey et al., 2012). More generally, active music making has been shown to promote wellbeing in old age (Hallam and Creech, 2016; Sheppard and Broughton, 2020) and the same holds for music listening (Laukka, 2007). Differences related to musical training have also been reported, although research on this variable is scarce (Greb et al., 2018). While musicians use music listening for being creative or forming their self-image (Tarrant et al., 2000), musically untrained adults listen to music with more emotion-related functions (Lehmann, 1993). Nevertheless, musicians also report using music for mood regulation (Saarikallio et al., 2020) and participation in activities such as singing and instrument playing is associated to socioemotional wellbeing (Krause et al., 2019). Perceived wellbeing through music performance or music listening is also positively related to individuals’ ratings of music’s importance in their lives (Saarikallio, 2012; Krause et al., 2019).

Contextual variables also influence the uses of music (North et al., 2004; Greb et al., 2018). Thus, being alone while listening to music contributes to relieving tension, coping with difficult times, enjoyment and reducing feelings of loneliness (Tarrant et al., 2000). Singing in group seems to be more beneficial to wellbeing than solo singing (Stewart and Lonsdale, 2016), which could be explained by the social gains of the choral activity (e.g., sense of support and community) (Livesey et al., 2012). Overcoming loneliness is predicted by contextual variables such as the activity done while listening (e.g., doing housework), the time of the day, and having the possibility of choosing the music to listen to Greb et al. (2018). Self-chosen music is also related to relaxation and enjoyment (Greasley and Lamont, 2011), which in turn is associated with background music while traveling (Heye and Lamont, 2010). Having more free time is linked to music making for emotional regulation (Saarikallio, 2011). Average level of attention to music and amount of time spent on music listening also relate to affect and wellbeing enhancement (Greasley and Lamont, 2011; Saarikallio, 2012; Greb et al., 2018). Concentration and clear goals during music performance predict positive affect experiences (Fritz and Avsec, 2007). Feelings of depression, anxiety and stress levels are related to goals such as diversion from worries, venting of negative emotions, entertainment, and maintenance and enhancement of positive mood (Thomson et al., 2014).

During the current COVID-19 crisis, research has already shown the usefulness of music to enhance wellbeing. Intervention through music listening has been proven to promote emotional wellbeing in Italian clinical staff by reducing feelings of fear, sadness, and worry (Giordano et al., 2020). In a survey study conducted in Australia, a positive association was found between music listening and life satisfaction (Krause et al., 2021). In a cross-cultural survey study in which respondents came from 11 different countries (United States, United Kingdom, Netherlands, Norway, Italy, Spain, Mexico, Brazil, Argentina, Colombia, China), music has been found to be more or as effective as other strategies (hobbies, physical activity, information-seeking, reading, eating/cooking, doing productive activities, mindfulness, movie watching) to fulfill different wellbeing-related goals (Granot et al., 2021). Similarly, another study has shown that, during the pandemic, after pooling together music listening and music playing, music is considered the most helpful coping activity in the United States, Italy, and Spain (Mas-Herrero et al., 2020). In addition, the more emotionally impacted by the crisis, the higher the reported levels of engagement in musical behavior and, in turn, the higher the levels of musical behavior, the less depressive symptoms experienced by individuals (Mas-Herrero et al., 2020). In another cross-cultural study conducted in the United Kingdom, the United States, France, Germany, Italy, and India, both personal and contextual variables have been found to predict the use of music for socioemotional coping during the crisis, including neuroticism and individuals’ importance of music among the former, and selecting different music compared to before the crisis and number of COVID-19 infections in the country among the latter (Fink et al., 2021). In Spain, a survey study has shown how adults of different ages have increased their use of music during the lockdown, with a positive impact in their perceived wellbeing (Cabedo-Mas et al., 2021). The influence of people’s employment situation during confinement was also analyzed and this contextual variable showed significant effects. As compared to prior to the crisis, furloughed individuals and teleworkers increased their time of music listening, while this was not observed in unemployed and face-to-face workers. Thus, being forced to spend more time at home led to more music consumption (Cabedo-Mas et al., 2021). This also had an impact on wellbeing perception. The furloughed and teleworkers were the groups who perceived the highest impact of music as a means to relaxing, escaping, improving their mood and generally coping better with the lockdown. The retirees were the ones who reported the lowest impact of music on their wellbeing. This was interpreted as a result of their older age. The elderly is the age group with the highest risk of death due to COVID-19, thus the group suffering from stricter social-distancing and lockdown measurements, and this vulnerability would diminish the positive impact of music on their emotional wellbeing (Cabedo-Mas et al., 2021).

Other contextual and personal variables could also influence the effect of music on emotional wellbeing during confinement, as would be expected when considering prior results on the relevance of personal and contextual variables both on the use of musical behaviors for affect regulation and wellbeing and the emotional effects of the lockdown in Spanish citizens. Thus, this research aimed to analyze the impact of the two types of variables (personal and contextual) on the perceived efficacy of music to fulfill wellbeing-related goals by adults during the strict COVID-19 lockdown established in Spain during March–June 2020. Given that the COVID-19 crisis is being maintained over time and that new lockdowns are being recurrently imposed, ascertaining which are the variables that are related to higher perceived wellbeing through musical behaviors in such an endangering situation is of relevance, since it may pave the way to clarifying who are the people who could profit the most from the use of music for wellbeing enhancement in the current pandemic. The data collected for this study are part of a larger cross-cultural survey on the use of music for wellbeing during COVID-19 times (Granot et al., 2021). Following prior literature (e.g., Greb et al., 2018; Krause et al., 2019; Luceño-Moreno et al., 2020; Sandín et al., 2020), among the personal variables we analyzed the effect of gender, age, musical training, personality traits, perceived importance of music, and resilience. COVID-19 context-related variables included living in a region with a high COVID-19 impact, being alone and having caring responsibilities during confinement, and perception of belonging to a risk group, since these variables have been linked to a reduction of individuals’ wellbeing during the lockdown in Spain (Balluerka-Lasa et al., 2020; Luceño-Moreno et al., 2020; Sandín et al., 2020). Time spent on music listening relates to affect and wellbeing, as previously mentioned (Greasley and Lamont, 2011; Saarikallio, 2012; Greb et al., 2018). Because of this, in the setting of this study, the amount of time of music listening as compared to prior to the crisis was also considered among the contextual variables. In the current research, the personal and contextual variables were the independent variables and the perceived efficacy of musical behavior for the achievement of different goals related to wellbeing were the dependent variables.

Although this was an exploratory study, we followed the reviewed literature to establish our hypotheses. Among the personal variables, we hypothesized that women would perceive a higher efficacy of musical behaviors as a means to improve their emotional wellbeing. This was based both on the fact that women have suffered a higher emotional impact during the COVID-19 lockdown in Spain (Justo-Alonso et al., 2020; Sandín et al., 2020) and on the results on the reported association between being women and the use of musical behavior for affect regulation (e.g., Chamorro-Premuzic et al., 2012; Krause et al., 2019). Research on the effect of age on the use of music for wellbeing enhancement has shown mixed results, as already seen. Therefore, we followed a prior study about the use of music during the COVID-19 crisis in Spain, in which the older adults reported the lowest impact of music on their wellbeing (Cabedo-Mas et al., 2021). Thus, we hypothesized that the younger adults would perceive a higher benefit of music for wellbeing. Musical experience is predictive of wellbeing (Krause et al., 2019) so we hypothesized that adults with musical training would perceive a higher efficacy of music for wellbeing improvement. Lower emotional stability as a personality trait is related to an emotional use of music (Chamorro-Premuzic et al., 2012) and music’s importance is associated to perceived wellbeing through musical behavior (Saarikallio, 2012; Krause et al., 2019). We hypothesized the same relationships in the context of our study. To our knowledge, there has been no prior study on the effect of resilience on the perceived efficacy of music for emotional wellbeing. However, it is known that a higher resilience protects from the negative emotional impact of the pandemic (Luceño-Moreno et al., 2020). Therefore, it was considered that if individuals with high resilience are less emotionally vulnerable, this feature would relate to a lower need of music as a means for wellbeing enhancement. Thus, we hypothesized that the higher the resilience, the lower the perceived efficacy of musical behaviors. We followed a similar reasoning regarding the COVID-19 contextual variables. During the lockdown in Spain, being in a region with high COVID-19 incidence, perception of being at risk, living alone, and having caring responsibilities have been associated to negative emotional consequences, as already mentioned (Balluerka-Lasa et al., 2020; Luceño-Moreno et al., 2020; Sandín et al., 2020). Considering that emotional strain is related to an emotional use of music (Vella and Mills, 2017), the higher emotional vulnerability linked to the aforementioned situations led us to hypothesize a significant association between the COVID-19 context-related variables and a higher perceived efficacy of music for emotional wellbeing. Finally, since a higher amount of music listening is related to an emotional use of music (Greasley and Lamont, 2011; Saarikallio, 2012), we hypothesized the same type of relationship regarding the listening time during the lockdown.

## Materials and Methods

### Participants

A total of 588 adults answered the online survey designed for the study. Inclusion criteria were to have answered at least 95% of the survey and to have been in lockdown in Spain during the time of strict confinement in this country. Thirty respondents failed to provide enough answers, 48 reported to have been abroad during the lockdown, and three reported not to have been in confinement. These participants were excluded from the study and thus the final sample was composed of 507 participants. The descriptive characteristics of the sample are presented in Table 1 (see also section “Materials”).

**TABLE 1:** Descriptive characteristics of the sample.

**Variable**	***N***	**%**
**Gender**		
Male	128	26.1
Female	362	73.9
**Age**		
18–24	69	13.8
25–34	117	23.4
35–54	252	50.5
55 or more	61	12.2
**Musical training**		
Without	242	48.7
1–3 years	80	16.1
4–9 years	60	12.1
More than 9 years	115	23.1
**Perception of belonging to a COVID-19 risk group**		
Not at all	151	30.3
To a very small or a small degree	188	37.3
To some degree	86	17.2
To a large or very large degree	74	14.8
**Living alone during lockdown**		
Yes	54	10.8
No	445	89.2
**Caring responsibilities**		
No	307	61.5
Yes, alone	36	7.2
Yes, shared with other people	156	31.3
**Regional COVID-19 impact during the lockdown**		
Lower	210	42.1
Higher	289	57.9

### Materials

The survey was part of a large-scale international research (Granot et al., 2021), as already mentioned. As such, it was a long survey in which different topics were evaluated. It included questions about the perceived efficacy of 10 different activities for wellbeing-related goals during the COVID-19 lockdown. Thus, the complete survey referred not only to the perceived efficacy of musical behavior but also to that of activities such as cooking or eating, physical exercise, movie watching, information seeking, working or doing other productive activities, reading, talking, spirituality or mindfulness. It also included sections about fears related to COVID-19, current mood of the respondent, current psychopathological symptoms as assessed with the Depression, Anxiety and Stress Scale (DASS-21) (Lovibond and Lovibond, 1995), activities done during the lockdown (e.g., use of online museums), personal music-related information (e.g., importance of music in life), and demographics. More details on the full survey can be found in Granot et al. (2021). Here we describe the data of the survey used for the current study. The questions included in this study are presented as Supplementary Material.

Four different emotional wellbeing goals were considered: diversion from the crisis, release and venting of negative emotions (e.g., stress, anxiety, and anger), enjoyment and maintaining good mood, and reducing loneliness and creating a sense of “togetherness.” As mentioned in the introduction, these are included among the socioemotional uses of music (e.g., Toyoshima et al., 2011; Sanal and Gorsev, 2014; Schäfer and Eerola, 2020), which explains why the former goals were analyzed in the study. For each of the goals, the perceived efficacy of musical behavior was analyzed through only one question. Specifically, participants were asked to assess how useful music was for them to fulfill each of the emotional wellbeing goals. Music referred here to listening, playing an instrument, or singing (i.e., any of these activities). A seven-point Likert scale was used for respondents to give their answer. Single questions have also been successfully used in the literature on music and affect regulation and wellbeing (Saarikallio et al., 2020; Krause et al., 2021). Considered as a scale, for these questions, Cronbach’s alpha was 0.84.

Information about the individual variables deemed relevant for the study was analyzed. As aforementioned, personal variables included gender, age, years of musical training, participants’ perception of music’s importance, personality, and resilience. The survey included a question about *participants’ age* in which respondents had to choose their corresponding age range. For this study, we considered four different age groups: 18–24, 25–34, 35–54, and older than 55. We followed prior studies in which the negative emotional consequences of the COVID-19 lockdown in Spain were compared across age groups (Sandín et al., 2020) and those in which age differences have been reported for the uses of music (Lonsdale and North, 2011). Participants were also asked whether they had experience in playing a musical instrument or singing and, if so, *how many years they were engaged in lessons or regular practice*. This musical experience was used as a measure of musical training. As with age, ranges of years were given as answers. Low, medium and high experience have been considered in prior work in which the effect of musical training on the uses of music was analyzed (Tarrant et al., 2000). It is also assumed that at least 10 years of training are required to consider someone as an expert or being able to attain the highest level of performance (Ericsson and Lehmann, 1996). Following this, for the musical training variable, we considered four categories: no musical training, 1–3 years of musical training, 3–9 years, and more than 9 years. Prior research has also used the number of years of musical engagement as a measurement of musical training or musical experience (Krause et al., 2019; Saarikallio et al., 2020). Participants with musical training were also asked to report the activity their musical experience referred to (i.e., specific musical instrument/s played and/or professional singing). The answers provided by respondents are shown in Table 2. In Spain, music education is compulsory within the educational system. However, music education is conceived as an element for personal and cultural development and is not focused on learning to play an instrument or doing professional singing. Therefore, this was here not considered as musical training.

**TABLE 2:** Music activities (percentage) reported by the musically trained respondents.

**Music activity**	**Musical training**		
	**1 – 3 years**	**4 – 9 years**	**More than 9 years**
Bow instruments	7.5	11.7	13.9
Plucked instruments	41.3	35	30.4
Woodwind instruments	2.5	26.7	27.8
Brass instruments	1.3	6.7	4.3
Percussion instruments	12.5	6.7	18.3
Piano or keyboard	30	30	42.6
Professional singing	7.5	18.3	13.9
Electronic instruments/music technology	2.5	0	9.6

*The data represent percentages within each musically trained group. Respondents indicated all the music activities their musical experience referred to.*

*Participants’ perception of music’s importance* was assessed with a single item through a 5-point Likert scale. The same has been successfully done in prior research (e.g., Saarikallio, 2012; Bonneville-Roussy et al., 2013; Krause et al., 2019). *Personality* was assessed by means of the Ten-Item Personality Inventory (TIPI) test in its Spanish version (Romero et al., 2012; Renau Ruiz et al., 2013). This test evaluates the traits of the Big-Five personality model (extraversion, agreeableness, conscientiousness, emotional stability and openness to experiences), shows good psychometric properties and is considered a good choice as a short personality measure in Internet-based studies (Romero et al., 2012; Renau Ruiz et al., 2013). The full scale is presented in Renau Ruiz et al. (2013). In our study, Cronbach’s alpha for extraversion, agreeableness, conscientiousness, emotional stability and openness to experiences were 0.73, 0.33, 0.46, 0.73, 0.45, respectively. These are very similar to the original data published in the studies in which the scale was validated for use in Spanish adults (Romero et al., 2012; Renau Ruiz et al., 2013) and also in the original scale published in English (Gosling et al., 2003). The unusually low internal consistency estimates as measured with Cronbach’s alpha are explained by the fact that only two items are included in each of the personality scales (Gosling et al., 2003). Because of this, test–retest reliability correlations are considered better estimates (Gosling et al., 2003) and, in fact, these have been considered appropriate both in the Spanish and the English versions of the scale (Gosling et al., 2003; Romero et al., 2012; Renau Ruiz et al., 2013). Considering the nature of our study, test-retest correlations were not here available. *Resilience* was assessed through the Spanish version of the 10-item Connor-Davidson Resilience Scale (10-item CD-RISC), which also has good psychometric properties and is a valid and reliable instrument for measuring resilience in adulthood (Notario-Pacheco et al., 2011; Serrano-Parra et al., 2013; Gras et al., 2019). The items included in this test can be seen in Notario-Pacheco et al. (2011). In our study, for this scale, Cronbach’s alpha was 0.84.

To evaluate the potential effect of the context experienced during the lockdown, participants were asked whether they *stayed alone during the lockdown*, whether they had *caring responsibilities*, and whether they *considered themselves belonging to a risk group of COVID-19*, i.e., their subjective perception of belonging to a risk group. COVID-19 risk perception has been related to negative affect (Luceño-Moreno et al., 2020; Yıldırım et al., 2021) and, conversely, health perceptions have a strong impact on wellbeing (Leite et al., 2019). This accounts for the relevance of choosing the aforementioned subjective perception measurement. Participants were also asked about the Spanish region where they had been in confinement. Two groups were afterward created for this latter variable according to the *regional COVID-19 impact during the lockdown* (higher vs. lower impact). Considering the accumulated percentage of people who died during the lockdown (from 14 March to 21 June 2020, as previously mentioned), the regions with higher impact were País Vasco (0.0185), Madrid (0.0056), Castilla y León (0.0014), Comunidad Valenciana (0.0014), Castilla La Mancha (0.0013), and Cataluña (0.0007) (Centro de Coordinación de Alertas y Emergencias Sanitarias del Ministerio de Sanidad de España, 2020). Through a 5-point Likert scale, participants also reported their *amount of time spent in music listening during the lockdown as compared to beforehand*. This type of single question has also been successfully used in previous studies (Saarikallio, 2012; Thomson et al., 2014).

### Procedure

The online survey was spread through Spanish university forums and social media. Care was taken to spread the survey not only in the general population but also among musicians, so that the potential effects of musical experience could be afterward studied. The survey was announced as a study focused on ascertaining the usefulness of music to deal with the negative emotions caused by the COVID-19 crisis in comparison with other possible means. The data were collected from the 6th of August to the 5th of December 2020. Most of the data were gathered in August (during summer holiday) and September (the return to work and school). The incidence in the first weeks of August was very low, but the rates of infections started to increase at the end of this month, originating the beginning of the second wave. This tendency was sustained for all the following weeks during which the data were gathered. The number of new deaths per million on 6th of August was 14, on 26th of September 148 (90% of the data were gathered till this day), and on 5th of December 225 (Thomas et al., 2020). Although there were restrictions during the time of data collection (e.g., facial coverings always required outside the home or restrictions on internal movement in same regions), the severe lockdown was over.

## Results

Most of the participants reported that musical behavior had helped them to achieve the four goals related to affect regulation and emotional wellbeing. Compared to individuals who answered that musical behavior was non-helpful or irrelevant for fulfilling the goals, a significantly higher number reported that music was of help. Thus, musical behavior was used as a strategy for Diversion from the crisis by 88.3% of respondents [χ^2^(1) = 196.22, *p* < 0.001], by 90.4% for Venting of negative emotions [χ^2^(1) = 251.22, *p* < 0.001], by 87.3% for Enjoyment and maintaining good mood [χ^2^(1) = 249.94, *p* < 0.001] and by 75.1% for Reducing loneliness and creating a sense of togetherness [χ^2^(1) = 93.75, *p* < 0.001].

To analyze the effect of each of the individual categorical variables deemed important in this study on the efficacy of music for the affect regulation and emotional wellbeing goals, one-way ANOVA and *t*-tests were used. To control for the Type I error rate in the ANOVA tests, *post hoc* analyses were conducted with *Tukey* or *Games-Howell*’s tests depending on the results of *Levene*’s tests for homoscedasticity.

When gender was considered, no significant differences were found on any of the goals (*p* > 0.05 in all cases). However, age was significant for two of them. Thus, there was a significant effect of age group on Diversion from the crisis [*F*(3,330) = 3.33, *p* = 0.02, *r* = 0.17] and on Enjoyment and maintaining good mood [*F*(3,445) = 4.14, *p* = 0.007, *r* = 0.16]. For Diversion from the crisis, significantly higher efficacy of musical behavior was found in the youngest group as compared to the oldest one [CI._95_ = 0.10 (lower) 1.31 (upper), *p* = 0.016]. A trend for the same effect was found in the youngest participants in comparison with both the participants aged 25–35 years [CI._95_ = −0.02 (lower) 0.93 (upper), *p* = 0.065] and those aged 35–54 [CI._95_ = 0.00 (lower) 0.81 (upper), *p* = 0.05]. No other between-group significant differences were found (*p* > 0.05). For Enjoyment and maintaining good mood, the music efficacy reported by the youngest participants was significantly higher than that reported by each of the other older age groups [18–24 vs. 25–34: CI._95_ = 0.03 (lower) 0.80 (upper), *p* = 0.03; 18–24 vs. 35–54: CI._95_ = 0.08 (lower) 0.71 (upper), *p* = 0.009; 18–24 vs. 55 or older: CI._95_ = 0.18 (lower) 1.28 (upper), *p* = 0.005]. No other between-groups significant differences were found (*p* > 0.05). As already mentioned, no significant effect of age group was found for Venting of negative emotions or for Reducing loneliness and creating a sense of togetherness (*p* > 0.05). Descriptive data together with a summary of these results are presented in Table 3.

**TABLE 3:** Music efficacy results for each goal by age and by musical training.

	**Diversion from the crisis**	**Venting of negative emotions**	**Enjoyment and maintaining good mood**	**Reducing loneliness and creating a sense of togetherness**
**Age**				
18–24	2.57 (0.93)	2.53 (0.75)	2.53 (0.76)	1.85 (1.47)
25–34	2.11^+^(1.23)	2.24 (1.05)	2.12*(1.16)	1.48 (1.23)
35–54	2.16^+^(1.11)	2.17 (1.09)	2.14**(1.11)	1.59 (1.22)
55 or more	1.83*(1.20)	2.10 (1.03)	1.80**(1.33)	1.83 (1.28)
**Musical training**				
Without	1.89 (1.15)	1.96 (1.12)	1.91 (1.18)	1.33 (1.21)
1–3 years	2.33*(0.96)	2.26 (0.95)	2.34**(0.89)	1.85*(1.16)
4–9 years	2.51**(0.84)	2.64***(0.69)	2.41**(0.92)	1.88*(1.20)
More than 9 years	2.48**(1.23)	2.62***(0.86)	2.40**(1.15)	2.00***(1.38)

*The scale used ranged from −3 to 3. Values in parentheses represent standard deviations.*

Comparisons refer to the youngest age group or to the group without musical training, respectively, for each of the variables. ^+^Trend at *p* < 0.10, *significant at *p* < 0.05, **significant at *p* < 0.01, ***significant at *p* < 0.001.

Musical training showed a significant effect on the efficacy of musical behavior for the four goals. For Diversion from the crisis [*F*(3,329) = 6.84, *p* < 0.001, *r* = 0.24], music efficacy was lower for the musically untrained participants compared with each of the musically trained groups [musically untrained vs. 1–3 years: CI._95_ = −0.84 (lower) −0.03 (upper), *p* = 0.03; musically untrained vs. 4–9 years: CI._95_ = −1.06 (lower) −0.19 (upper), *p* = 0.002; musically untrained vs. more than 9 years: CI._95_ = −1.01 (lower) −0.15 (upper), *p* = 0.003]. No other between group significant differences were obtained. Similar results were found for Venting of negative emotions [*F*(3,380) = 11.21, *p* < 0.001, *r* = 0.29]. The efficacy of musical behavior was significantly lower for musically untrained participants as compared to those with at least 4 years of musical training [musically untrained vs. 4–9 years: CI._95_ = −1.03 (lower) −0.33 (upper), *p* < 0.001; musically untrained vs. more than 9 years: CI._95_ = −0.98 (lower) −0.33 (upper), *p* < 0.001] but no significant differences were found between the musically untrained group and that with 1–3 years of musical training [CI._95_ = −0.66 (lower) 0.07 (upper), *p* = 0.15]. For Enjoyment and maintaining good mood [*F*(3,443) = 6.97, *p* < 0.001, *r* = 0.21], music efficacy was significantly lower for the musically untrained participants in all cases [musically untrained vs. 1–3 years: CI._95_ = −0.77 (lower) −0.09 (upper), *p* = 0.007; musically untrained vs. 4–9 years: CI._95_ = −0.88 (lower) −0.11 (upper), *p* = 0.007; musically untrained vs. more than 9 years: CI._95_ = −0.85 (lower) −0.14 (upper), *p* = 0.003]. The same was found for Reducing loneliness and creating a sense of togetherness [*F*(3,367) = 7.30, *p* < 0.001, *r* = 0.24; musically untrained vs. 1–3 years: CI._95_ = −1.00 (lower) −0.05 (upper), *p* = 0.025; musically untrained vs. 4–9 years: CI._95_ = −1.10 (lower) −0.01 (upper), *p* = 0.04; musically untrained vs. more than 9 years: CI._95_ = −1.10 (lower) −0.25 (upper), *p* < 0.001]. Descriptive data together with a summary of these results are shown in Table 3.

None of the COVID-19 context-related variables had significant effects (living in a region with a high COVID-19 impact, perception of belonging to a risk group, being alone, having caring responsibilities: *p* > 0.05 in all cases).

Relationships between the efficacy of music for the wellbeing goals and music’s importance, time spent on music listening during lockdown as compared to the times before the crisis, personality traits, and resilience were analyzed by means of Pearson’s correlations. As shown in Table 4, music efficacy for the four goals was significantly positively correlated with music’s importance and time spent on music listening. Considering the personality traits, only openness to experience was significantly positively correlated to Venting of negative emotions and Enjoyment and maintaining good mood. Resilience was not significantly correlated to music’s efficacy for any of the wellbeing goals. The lack of significance of resilience was unexpected. It has been suggested that lower resilience could explain why the negative emotional consequences of the lockdown are higher in younger than in older adults (Justo-Alonso et al., 2020). To test whether this was really the case, another one-way ANOVA test was conducted with resilience as dependent variable and age as independent variable. A significant effect was found [*F*(3,494) = 5.72, *p* = 0.001, *r* = 0.18]. *Tukey*’s *post hoc* tests revealed that the youngest adults had lower resilience (*M* = 26.25, *SE* = 5.91) than the ones aged 35–54 (*M* = 29.28, *SE* = 5.68) or more than 55 (*M* = 29.72, *SE* = 6.33) [CI._95_ = −5.09 (lower) −0.97 (upper), *p* = 0.001; CI._95_ = −6.29 (lower) −0.96 (upper), *p* = 0.003, respectively]. No other between group significant differences were found (*p* > 0.05).

**TABLE 4:** Pearson’s correlations between music efficacy for the affect-regulation goals, music’s importance, time spent on music listening and personality traits.

	**Diversion from the crisis**	**Venting of negative emotions**	**Enjoyment and maintaining good mood**	**Reducing loneliness and creating a sense of togetherness**
Music’s importance	0.57**	0.59**	0.52**	0.40**
Time spent on music listening	0.32**	0.30**	0.33**	0.28**
Extraversion	–0.04	–0.01	–0.04	0.02
Agreeableness	0.09	0.08	0.04	0.02
Conscientiousness	–0.07	–0.02	–0.02	0.02
Emotional stability	0.05	–0.03	–0.03	0.05
Openness to experiences	0.11	0.14**	0.16**	0.09
Resilience	0.04	0.03	0.06	0.09

**Significant at *p* < 0.01, *significant at *p* < 0.05.

We, then, analyzed which of the variables that had shown any significant effect on music efficacy for fulfilling the goals could be predictive of such an efficacy. For this, musical training (dummy coded), music’s importance and time spent on music listening during lockdown were entered as predictors in four different models (one for each goal) by using backward stepwise regression. Age (dummy coded) was also included for the aims in which this variable had shown significance (Diversion from the crisis, Enjoying and maintaining good mood). For the same reason, for Venting of negative emotions and Enjoyment and maintaining good mood, openness to experience was also included in the models. Significant models were found for each of the goals [Diversion from the crisis: *F*(3,329) = 59.35, *p* < 0.001, *R*^2^ = 0.35; Venting of negative emotions: *F*(3,380) = 73.91, *p* < 0.001, *R*^2^ = 0.37; Enjoyments and maintaining good mood: *F*(2,444) = 95.51, *p* < 0.001, *R*^2^ = 0.30; Reducing loneliness and creating a sense of togetherness: *F*(2,368) = 42.07, *p* < 0.001, *R*^2^ = 0.19]. Music’s importance and the amount of time spent in music listening during the lockdown were significant predictors of music efficacy on the four goals. Age (18–24 vs. 55 or more) only showed to be a significant predictor for Diversion from the crisis. Musical training significantly predicted music efficacy only for Venting of negative emotions, but only for the comparison between musically untrained and 4–9 years. Openness was not included as a predictor in any of the models. Results are presented in Table 5.

**TABLE 5:** Regression models of music efficacy on the affect-regulation goals.

**Predictors**	***B***	***SE B***	**β**	**Unique variance (%)**
**Diversion from the crisis**				
Music’s importance	0.59	0.05	0.52	24.01
Amount of music listening	0.18	0.06	0.14	1.69
Age (18–24 vs. 55 or more)	–0.75	0.35	–0.10	1.00
**Venting of negative emotions**				
Music’s importance	0.58	0.05	0.55	27.04
Amount of music listening	0.13	0.05	0.11	1.00
Musical training (musically untrained vs. 4–9 years)	0.27	0.14	0.08	0.64
**Enjoyment and maintaining good mood**				
Music’s importance	0.51	0.05	0.46	18.49
Amount of music listening	0.25	0.06	0.19	3.24
**Reducing loneliness and creating a sense of togetherness**				
Music’s importance	0.44	0.06	0.35	10.89
Amount of music listening	0.24	0.07	0.16	2.56

*SE, standard error. Unique variance refers to the percentage of variance explained by the predictor after controlling for the effect of the remaining predictors included in the model.*

Musical training and age were thus significant predictors in only two goals, respectively, even though prior ANOVA results had shown other significant effects, as previously reported. Taking this into account, together with the fact that both music’s importance and the amount of time spent in music listening were significant predictors of the efficacy of music for all the goals, we considered it important to analyze possible age and musical training between-group differences in these latter variables. One-way ANOVA tests, with *Games–Howell*’s tests for *post hoc* analyses, were again used for this aim. A significant effect of age was found on music’s importance [*F*(3,495) = 8.81, *p* < 0.001, *r* = 0.263]. Music was considered more important for the youngest participants as compared to any of the other older groups [18–24 vs. 25–34: CI._95_ = 0.12 (lower) 0.82 (upper), *p* = 0.004; 18–24 vs. 35-54: CI._95_ = 0.30 (lower) 0.95 (upper), *p* < 0.001; 18–24 vs. 55 or older: CI._95_ = 0.33 (lower) 1.31 (upper), *p* < 0.001], and no other between-group significant differences were found (*p* > 0.05). Musical training also showed a significant effect on music’s importance [*F*(3,493) = 28.77, *p* < 0.001, *r* = 0.39]. Musically untrained participants reported lower importance of music in comparison with each of the musically trained groups [musically untrained vs. 1–3 years: CI._95_ = −0.75 (lower) −0.08 (upper), *p* = 0.008; musically untrained vs. 4–9 years: CI._95_ = −0.97 (lower) −0.22 (upper), *p* < 0.001; musically untrained vs. more than 9 years: CI._95_ = −1.23 (lower) −0.75 (upper), *p* < 0.001]. In addition, participants with the longest musical training reported higher importance of music as compared to the other two musically trained groups [more than 9 years vs. 1–3 years: CI._95_ = 0.25 (lower) 0.90 (upper), *p* < 0.001; more than 9 years vs. 4-9 years: CI._95_ = 0.02 (lower) 0.76 (upper), *p* = 0.034]. No significant differences were found between participants with 1–3 years of musical training and those with 4–9 years of music experience (*p* > 0.05). Unlike for music’s importance, no significant effect of age or musical training was found on the amount of time spent in music listening (*p* > 0.05). Descriptive results for the two music variables are shown in Table 6.

**TABLE 6:** Importance of music and Time spent in music listening by age and by musical training.

	**Importance of music**	**Time spent in music listening**
**Age**		
18–24	4.48 (0.87)	3.97 (0.84)
25–34	4.01**(0.93)	3.80 (0.92)
35–54	3.85***(1.04)	3.79 (0.88)
55 or more	3.66***(1.22)	3.64 (0.90)
**Musical training**		
Without	3.59 (1.07)	3.71 (0.90)
1–3 years	4.00**/(0.97)	3.81 (0.93)
4–9 years	4.18***/***(0.98)	4.05 (0.89)
More than 9 years	4.57***/*(0.66)	3.83 (0.81)

The scale ranged from 1 to 5. Values in parentheses represent standard deviations. Comparisons refer to the youngest age group for the age variable. For musical training, to the left of the “/” sign, comparisons refer to the groups without musical training; to the right, they refer to the more than 9 years group. *Significant at *p* < 0.05, **significant at *p* < 0.01, ***significant at *p* < 0.001.

**FIGURE 1:**
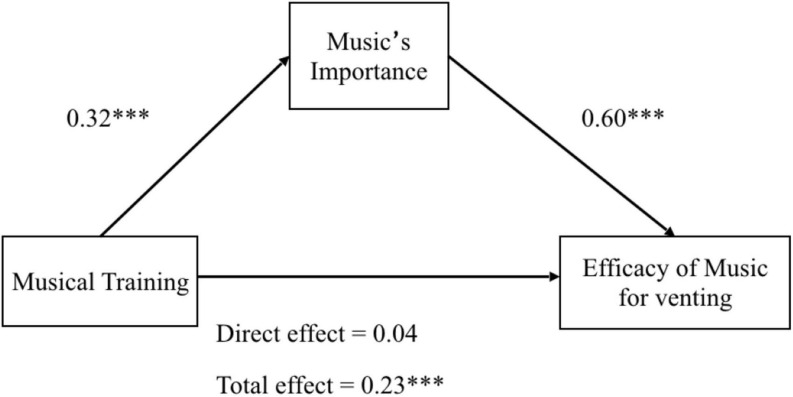
Model of the effect of musical training on the perceived efficacy of music for venting of negative emotions, mediated by music’s importance. p < 0.001^∗∗∗^.

Considering the latter ANOVA results, mediation analyses were performed to assess the possible mediating role of music’s importance on the linkage between musical training and the perceived efficacy of music in obtaining the different wellbeing goals. We used Preacher and Hayes’ (2004) bootstrapping method in which the unstandardized indirect effects were computed for each of 10,000 bootstrapped samples, and the 95% confidence interval was computed by determining the indirect effects at the 2.5th and 97.5th percentiles. The results revealed that the total effect of musical training on the perceived efficacy of music was significant (see Table 7) for all the goals. With the inclusion of the mediating variable music’s importance, the impact of musical training on the perceived efficacy of music became non-significant, again for all the goals. The indirect effect of musical training on the perceived efficacy of music through music’s importance was found significant in all the goals as well. This shows that the relationship between musical training and the perceived efficacy of music is fully mediated by the level of music’s importance perceived by participants. To illustrate this relationship, Figure 1 shows that the standardized regression coefficient between musical training and music’s importance was statistically significant [*b* = 0.32, CI._95_ = 0.24 (lower) 0.43 (upper), *p* < 0.001], as was the standardized regression coefficient between music’s importance and the perceived efficacy of music for venting negative emotions [*b* = 0.60, CI._95_ = 0.50 (lower) 0.69 (upper), *p* < 0.001]. The coefficient between music’s importance and the perceived efficacy of music was also highly significant for diversion [*b* = 0.65, CI._95_ = 0.54 (lower) 0.76 (upper), *p* < 0.001], enjoyment [*b* = 0.59, CI._95_ = 0.49 (lower) 0.69 (upper), *p* < 0.001], and reducing loneliness [*b* = 0.47, CI._95_ = 0.34 (lower) 0.59 (upper), *p* < 0.001].

**TABLE 7:** Mediation analysis of the effect of musical training on perceived efficacy of music in obtaining wellbeing goals, mediated by music’s importance.

	**Total effect c (MT→EM)**		**Direct effect c’ (MT→EM)**		Indirect effect a*b (MT→MI→EM)	
	**Estimate (*SE*)**	**CI**	**Estimate (*SE*)**	**CI**	**Estimate (*SE*)**	**CI**
Diversion	0.21 (0.05)	0.11, 0.30	−0.00 (0.04)	−0.09, 0.08	0.21 (0.03)	0.15, 0.27
Venting	0.23 (0.04)	0.15, 0.32	0.04 (0.04)	−0.02, 0.12	0.19 (0.03)	0.14, 0.24
Enjoyment	0.17 (0.04)	0.09, 0.26	−0.02 (0.04)	−0.09, 0.06	0.19 (0.02)	0.14, 0.24
Reducing loneliness	0.23 (0.05)	0.13, 0.33	0.08 (0.05)	−0.03, 0.18	0.15 (0.02)	0.11, 0.21

*MT, musical training; EM, efficacy of musical behaviors; MI, music’s importance; CI, Confidence Interval.*

Additional mediation analyses were carried with age as a predictor of the perceived efficacy of music, mediated again by the importance attributed to music by the participants. The analyses were carried for the two goals in which significant differences between age groups had been previously found (i.e., diversion and enjoyment). Age was found to be a significant predictor of the perceived efficacy of music in both the case of diversion [*b* = −0.18, CI._95_ = −0.34 (lower) −0.04 (upper), *p* < 0.001] and enjoyment [*b* = −0.18, CI._95_ = −0.30 (lower) −0.06 (upper), *p* < 0.01] when the mediator was not included. The regression between age and the mediator (music’s importance) was significant for diversion [*b* = −0.22, CI._95_ = –0.34 (lower) −0.10] and enjoyment [*b* = −0.24, CI._95_ = −0.35 (lower) −0.16 (upper), *p* < 0.001]. The regression between the mediator and the perceived efficacy of music was significant for both diversion [*b* = 0.64, CI._95_ = 0.54 (lower) 0.74 (upper), *p* < 0.001] and enjoyment [*b* = 0.57, CI._95_ = 0.48 (lower) 0.66 (upper), *p* < 0.001]. When controlling for music’s importance, i.e., the mediator, age was a non-significant predictor for both goals: diversion [*b* = −0.04, CI._95_ = –0.16 (lower) 0.07 (upper), *p* > 0.05] and enjoyment [*b* = −0.05, CI._95_ = −0.15 (lower) 0.06 (upper), *p* > 0.05]. Therefore, it was found that music’s importance fully mediated the relationship between age and the perceived efficacy of music.

## Discussion

Musical behaviors were used by a very large amount of Spanish people to fulfill different goals related to emotional wellbeing. This perception of the value of music has already been reported for the Spanish society (Mas-Herrero et al., 2020; Cabedo-Mas et al., 2021). The perceived efficacy of this strategy nevertheless varied according to personal variables. Between-groups significant differences were found as a function of age. The youngest participants reported higher efficacy of music in obtaining feelings of enjoyment and maintaining their good mood and to get distracted from the crisis and this is consistent with our hypotheses. Under normal everyday life circumstances, older adults have been reported to be less likely to listen to music to regulate their mood, which has been explained by the fact that older people may be more adept at managing their emotions (Lonsdale and North, 2011). In the current COVID-19 crisis, the older the person, the higher the risk of getting infected and, in the case of the elderly, this has translated into especially severe social distancing measurements, which may have made them more vulnerable to the negative emotions that have come as a consequence of the lockdowns. However, reports on the psychopathology experienced by the Spanish population during the confinement have shown that young adults are actually more vulnerable than older people (González-Sanguino et al., 2020; Justo-Alonso et al., 2020; Sandín et al., 2020). It has been suggested that this may be explained by a lower resilience in the younger adults (Justo-Alonso et al., 2020). We found this pattern in our study. In addition, the lockdown largely disrupted the habits of the youngest adults who suddenly had to spend all their time at home with their main activities interrupted; thus, a larger change in their everyday routines could account for the higher vulnerability of the younger adults (Justo-Alonso et al., 2020). Although no significant differences were found among the age groups regarding the perceived amount of music listened to during the lockdown as compared to prior times, generally, young adults spend more time engaged with music in their everyday lives (Lonsdale and North, 2011). All of this could explain why this group reported a higher efficacy of music on the promotion of their wellbeing. However, younger and older adults perceived a similar rate of music efficacy to reduce loneliness. This could be accounted for by the fact that loneliness during confinement was a concern that has been reported by both young and older Spanish people (Justo-Alonso et al., 2020).

Among the personal variables studied in this research, unlike what we had hypothesized, gender did not show any significant effect. Other research has reported that women are more likely than men to use music with an emotional function (Lonsdale and North, 2011; Chamorro-Premuzic et al., 2012; Ter Bogt et al., 2017). They also perceive larger wellbeing benefits linked to music participation (Krause et al., 2019). Therefore, our results are unexpected, at least to some extent, since other studies have also shown lack of gender differences in the emotional use of musical behaviors (Livesey et al., 2012; Saarikallio, 2012). Research has shown that, during the pandemic, the more emotionally vulnerable, the higher the level of music-related activities (Mas-Herrero et al., 2020). Although the negative emotional consequences of the COVID-19 lockdown experienced in Spain have been higher in women, men have also suffered from strong negative emotions and their patterns of emotional problems have been very similar to those observed in women (Sandín et al., 2020). The increase of negative emotions in men may have canceled out the gender differences that would have been expected according to the extant literature. Thus, the specific circumstances of the COVID-19 crisis would be responsible for the lack of a gender effect.

Between-group significant differences on the efficacy of music for the fulfillment of the wellbeing goals were found as a function of musical training. The musically trained adults (51.3% of respondents) reported higher music efficacy than their musically untrained counterparts. Prior research has shown that while musically untrained adults tend to listen to music with an emotional function, individuals with musical training are more likely to use music listening for eudemonic reasons (Lehmann, 1993; Tarrant et al., 2000). However, other studies have found that musicians use music for affect regulation (Saarikallio et al., 2020) and that music making is associated to a higher perception of wellbeing (Krause et al., 2019). Along the same lines, our research shows that under stressful circumstances such as those experienced during the COVID-19 lockdown in Spain, musical training can enhance the usefulness of music for wellbeing promotion. Interestingly, this effect was seen at any level of musical training. Thus, even in the group with only 1–3 years of musical training, the efficacy of music activities was considered higher than that in the musically untrained group. Considering the paucity of research on the effects of musical training for the uses of music, these are important results. To our knowledge, this is the first study directly comparing musically trained and untrained adults’ perceived efficacy of music for wellbeing enhancement.

Among the personality traits, only openness was significantly related to wellbeing through the goals of venting of negative emotions and enjoyment and maintaining good mood. Unlike what we had hypothesized, no significant correlation was found between emotional stability and the efficacy of music for emotional wellbeing and this is inconsistent with prior results found internationally, including Spain (Chamorro-Premuzic et al., 2009a, b, 2012; Fink et al., 2021). The relationship between emotional stability and the emotional use of music has been explained by considering individual differences associated with negative affect (Chamorro-Premuzic et al., 2009a). In the context of the lockdown established in Spain as a consequence of the COVID-19 crisis, negative emotions were widespread (Justo-Alonso et al., 2020; Sandín et al., 2020) and this may contribute to explaining the lack of a significant relationship between emotional stability and the emotional use of music. Openness to experience has been related to the use of music for enriching experiences (Chamorro-Premuzic et al., 2009a, 2012). In the Spanish society, during the lockdown, people searched for new groups and music styles as a means to create pleasure and stimulation, thus creating positive experiences through music (Cabedo-Mas et al., 2021). This may account for the relationship found between openness to experience and the efficacy of musical behaviors for wellbeing enhancement. Although unexpected, this result is not specific to Spain, having been found cross-culturally (Granot et al., 2021).

Considering that low emotional stability has been previously related to a higher use of music for affect regulation (Chamorro-Premuzic et al., 2009a, 2012; Vella and Mills, 2017) and the protective role of resilience on negative emotional symptoms (Luceño-Moreno et al., 2020), a relationship between lower resilience and an emotional use of music was expected. However, such an association was not found either. Just as suggested regarding emotional stability, it may be that this result is explained by the generalized impact of the pandemic on individuals’ mood. Thus, resilience, although a personal and intrinsic feature, is also a dynamic psychological factor that can be malleable by the context (Román-Mata et al., 2020). To our knowledge, this is the first study which analyzes the relationship between resilience and the efficacy of musical behavior for wellbeing enhancement. Future research should study the potential association in other stressful situations. As previously mentioned, it should also be considered that our results support the view that, in the current pandemic, the younger Spanish adults have lower resilience than the older ones, being this a factor contributing to explaining their higher levels of psychopathology (Justo-Alonso et al., 2020). In turn, and as aforementioned, this may be related to their higher perception of the efficacy of music for emotional wellbeing improvement.

None of the COVID-19 context-related variables (i.e., living in a region with a high COVID-19 impact, perception of belonging to a risk group, and being alone and having caring responsibilities during confinement) showed any statistical effect on the efficacy of music for the fulfillment of the wellbeing goals. On the contrary, prior research did show differences in the perceived value of music for wellbeing promotion as a function of the employment situation during the lockdown established in Spain (Cabedo-Mas et al., 2021). The benefit of music has been rated higher for the adults who were teleworking or furloughed during confinement, which could be explained by the larger disruption imposed by the lockdown in their everyday routines (Cabedo-Mas et al., 2021). This is similar to what we have reported for the youngest adults in this study. Therefore, disruption in everyday life seems to be a relevant factor mediating the perceived effect of music on emotional wellbeing during confinement. Although all the COVID-19 context-related variables included in this research were potentially endangering adults’ emotional health, none of them were directly related to routine’s disruption. This may explain their lack of significant effects. Along the same lines, other research has suggested that it is the everyday life disruption caused by the lockdowns, and not the COVID-19 incidence rates, what leads individuals to choose specific music patterns with the aim of reducing negative affect (Yeung, 2020).

The perceived amount of time devoted to music listening during the lockdown as compared to beforehand was the only contextual variable with a significant effect. It was also a significant predictor of the efficacy of music for the wellbeing goals. However, its effect was small. Among the variables included in this study, music’s importance in one’s life was the main predictor of the efficacy of music for all the emotional wellbeing goals. Thus, the unique variance explained by music’s importance showed medium or even large effects. Musical training and age also contributed to predicting music’s efficacy for wellbeing in a unique way (for venting of emotions and diversion from the crisis, respectively). However, the effect of these variables was also small. In the case of musical training, it should be noted that although between-group significant differences had been previously found for all the wellbeing goals, this variable was a significant predictor of only one of them. Participants’ perception of music’s importance (but not perceived amount of music listening) was higher in the musically trained than in the musically untrained adults. In this light, mediation analyses showed that the effect of musical training on the perceived efficacy of musical behaviors for each of the wellbeing goals is actually indirect. It is through the musically trained adults’ higher perception of music’s importance that musical training influences the perceived efficacy of music. The higher the musical training, the higher the importance attached to music in life and the stronger its effects on the person. The same mediation effect was found regarding age. The youngest adults perceived that music was more important for them and age showed an indirect predictive role on the perceived efficacy of musical behaviors for diversion from the crisis and enjoyment. The relevance of music’s importance as a predictor of wellbeing within the context of music participation is consistent with prior research (Krause et al., 2019). It is also consistent with the results of another study focused on the current pandemic (Fink et al., 2021).

Although this study has shown relevant results, we should also note its limitations. First, the information was collected retrospectively when the severe lockdown in Spain was already over and this may introduce confounds related to memory. These possible confounds are notable for the question referred to the amount of time spent on listening to music as compared to prior to the crisis. Considering the effects of subjective perceptions on wellbeing (Leite et al., 2019), more than factual data, we were interested in the perceived estimation of music listening time. Even though, the question required participants not only to think of their music listening time during the lockdown when it was already over, but also to compare this time with their habits before the pandemic. Although this is a procedure also followed in other research on the uses of music during COVID-19 times (Mas-Herrero et al., 2020), the results concerning this question should be taken with caution. Second, the possible distress experienced during the lockdown was not evaluated in the survey, even though the levels of stress, anxiety and depression -among other negative emotional consequences of the COVID-19 crisis- could modulate the effects of the variables studied in this research. It should also be highlighted that the correlational nature of this research prevents from drawing any conclusions on the possible causality of music activities on wellbeing promotion. In addition, in this study, the number of musically trained participants was very high because we deliberately distributed the survey among musicians’ nets in order to study the effect of musical training on the perceived efficacy of musical behavior for wellbeing enhancement. Although the results in this respect represent a relevant contribution given the paucity of research on this topic, having such a high rate of musically trained adults makes the sample not to be fully representative of the Spanish society. Even though, the sample was large enough so that the two groups of respondents (i.e., musically trained and untrained adults) may be considered representative of their corresponding groups. The percentage of female respondents was also much higher than that of male respondents. However, this is a known problem very often found in survey studies in general (e.g., Justo-Alonso et al., 2020; Sandín et al., 2020) and in studies on the effects of music-related activities on emotional wellbeing in particular (e.g., Livesey et al., 2012; Krause et al., 2019; Saarikallio et al., 2020; Sheppard and Broughton, 2020). We should also note that this study only focused on adults. Including children would have also been of interest, given that they have also suffered due to the confinement (Giménez-Dasí et al., 2020).

The changing nature of the COVID-19 crisis which, unfortunately, is advancing through different waves worldwide makes it likely that new lockdowns will need to be imposed by the governments, as indeed is already happening (Thomas et al., 2020). All of this warrants the need of further research on the variables that modulate the efficacy of music for the enhancement of emotional wellbeing within the current crisis. Among these, studying potential differences between music listening and music performance would be of relevance, a distinction that was not done in our study. Since the survey was long, to guarantee a high rate of response, the two types of activities were collapsed. In this way, we considered musical behavior as a unitary construct. This is common practice in the field and even instruments developed to evaluate the functional uses of music-related activities not always do the distinction (Saarikallio, 2008, 2012). It underlies the idea that the two musical behaviors can fulfill the same socioemotional functions (Saarikallio, 2011); they also share many features as musical processes, to the extent that the reciprocal-feedback model of musical response used here as a framework to study the efficacy of musical behavior to fulfill socioemotional aims is applicable to both music perception and production (Hargreaves, 2012; Hargreaves et al., 2016). Furthermore, as reviewed in the introduction, when analyzing the potential effects of personal and contextual variables on the uses of music-related activities for emotional wellbeing enhancement (i.e., the aim of this study), the same pattern of results is found. In the context of the COVID-19 crisis, both music listening and music making contribute to affect regulation and each of these explains a very similar amount of variance in socioemotional coping (Fink et al., 2021). However, there is place for potential differences. For example, music listening is more often used for affect regulation even in individuals who play musical instruments (Saarikallio, 2012), but the benefits of music making may be higher than those of music listening (Raglio et al., 2015). From our study, we cannot make any distinctions between music perception and music production and this represents another limitation. Future research should evaluate whether the effects of the personal and contextual variables here studied are different when separately considering music listening, singing and instrument playing. For this, it would be better if the study were conducted online and not retrospectively, to avoid any potential bias linked to memory-based answers.

Musical behaviors have been used by the Spanish society to fulfill emotional wellbeing goals in the context of the COVID-19 lockdown established in Spain during March–June 2020. This study has contributed to ascertaining which are the variables that modulate the efficacy of music activities for the achievement of such goals. Among the variables included in this research, the personal ones showed the highest impact on music’s efficacy. The youngest adults and those who had musical training reported the highest efficacy of music activities for wellbeing enhancement, and music’s importance was found to be the main significant predictor of music’s perceived efficacy. Our findings suggest that the people who have been reported to be emotionally more vulnerable during the lockdown due to the strong impact of this situation on their everyday routines or to their lower resilience perceive a higher benefit from music. Being musically trained, even for a small number of years, seems to lead to a perception of higher efficacy of music for the achievement of emotion-related wellbeing goals. However, this effect is explained by the musically trained individuals’ higher perception of music’s importance. Although musical behavior can be generally considered as important for wellbeing enhancement, our study highlights who are the people who could benefit the most from musical activities for such an aim, at least within the current context of the COVID-19 crisis.

## Data Availability Statement

The raw data supporting the conclusions of this article will be made available by the authors, without undue reservation.

## Ethics Statement

The studies involving human participants were reviewed and approved by the Faculty of Humanities’ Research Ethics Committee, the Hebrew University of Jerusalem (Israel). The participants provided their written informed consent to participate in this study.

## Author Contributions

PM-C, IG-B, DS, and RG designed the study. PM-C and IG-B collected the data. PM-C and DS analyzed the data. PM-C wrote the first draft. All the authors contributed to manuscript revision, read, and approved the submitted version.

## Conflict of Interest

The authors declare that the research was conducted in the absence of any commercial or financial relationships that could be construed as a potential conflict of interest.
