# Supplementary material for: The Efficacy of Music for Emotional Wellbeing During the COVID-19 Lockdown in Spain: An Analysis of Personal and Context-Related Variables
Source: Front Psychol. 2021 Apr 9;12:647837. doi: 10.3389/fpsyg.2021.647837 (PMC8062927; doi:10.3389/fpsyg.2021.647837)
Supplement: Supplementary file 1 [file Data_Sheet_1.docx]

**Supplementary Material.**

**Questions included in the study (Spanish version)**

**… En qué grado han sido una ayuda o un obstáculo para conseguir los objetivos:**

**El objetivo: Desahogarme y liberar las emociones negativas (por ejemplo, estrés, ansiedad o enfado).**

Escuchar o hacer música (tocando un instrumento musical o cantando)

| Un gran obstáculo | Un obstáculo | Un pequeño obstáculo | Irrelevante | Una pequeña ayuda | Una ayuda | Una gran ayuda |
| --- | --- | --- | --- | --- | --- | --- |
| -3 | -2 | -1 | 0 | 1 | 2 | 3 |

**El objetivo: Evadirme de la crisis por la COVID-19**

Escuchar o hacer música (tocando un instrumento musical o cantando)

| Un gran obstáculo | Un obstáculo | Un pequeño obstáculo | Irrelevante | Una pequeña ayuda | Una ayuda | Una gran ayuda |
| --- | --- | --- | --- | --- | --- | --- |
| -3 | -2 | -1 | 0 | 1 | 2 | 3 |

**El objetivo: Divertirme y mantener el buen humor.**

Escuchar o hacer música (tocando un instrumento musical o cantando)

| \| Un gran obstáculo \| Un obstáculo \| Un pequeño obstáculo \| Irrelevante \| Una pequeña ayuda \| Una ayuda \| Una gran ayuda \| \| --- \| --- \| --- \| --- \| --- \| --- \| --- \| \| -3 \| -2 \| -1 \| 0 \| 1 \| 2 \| 3 \| |  |  |  |  |  |  |
| --- | --- | --- | --- | --- | --- | --- | --- | --- | --- | --- | --- | --- | --- | --- | --- | --- | --- | --- | --- | --- |

**El objetivo: Reducir la soledad y sentirme más unido/a a la gente**

Escuchar o hacer música (tocando un instrumento musical o cantando)

| \| Un gran obstáculo \| Un obstáculo \| Un pequeño obstáculo \| Irrelevante \| Una pequeña ayuda \| Una ayuda \| Una gran ayuda \| \| --- \| --- \| --- \| --- \| --- \| --- \| --- \| \| -3 \| -2 \| -1 \| 0 \| 1 \| 2 \| 3 \| |  |  |  |  |  |  |
| --- | --- | --- | --- | --- | --- | --- | --- | --- | --- | --- | --- | --- | --- | --- | --- | --- | --- | --- | --- | --- |

**En general, ¿cómo de importante es la música para ti?**

- No me importa.
- Me importa poco
- Me importa bastante
- Es muy importante para mí
- Es extremadamente importante para mí

**¿Cuánto tiempo has dedicado a escuchar música durante el confinamiento en comparación con el tiempo anterior a la crisis?**

- Mucho menos
- Menos
- El mismo
- Más
- Mucho más

**¿Cuántos años tienes?**

- 18
- 18 - 24
- 25 - 34
- 35 - 44
- 45 - 54
- 55 - 64
- 65 - 74
- 75 - 84
- Más de 85

**¿Cuál es tu género?**

- Hombre
- Mujer
- Prefiero no contestar
- Prefiero describirme yo

**¿Con quién has pasado el confinamiento?**

- Solo
- Con mi pareja
- Con mi núcleo familiar (uno o dos adultos con hijos)
- Con mi familia, que incluye a varias generaciones (abuelos, padres, nietos, …)
- Con amigos con los que no suelo vivir
- Con compañeros de piso
- No he estado confinado/a

**¿Tenías que cuidar de alguien?**

- No
- Sí, he cuidado de los niños yo solo/a
- Sí, he cuidado de los niños, pero no solo/a.
- Sí, he cuidado yo solo/a de un familiar o amigo.
- Sí, he cuidado de un familiar o amigo, pero no solo/a.

**¿Dónde has pasado el confinamiento?**

- Comunidad de Madrid
- Cataluña
- Castilla y León
- Castilla la Mancha
- País Vasco
- Comunidad Valenciana
- Otras comunidades en España
- En el extranjero. Escribe el país en el que has estado

**¿En qué medida crees que perteneces a uno de los grupos de riesgo frente al virus COVID-19?**

- Para nada
- Muy poco
- Poco
- Algo
- Bastante
- Seguramente

**¿Tienes experiencia tocando un instrumento musical o cantando? En caso afirmativo, ¿durante cuántos años has asistido a clases y/o has practicado con regularidad?**

- No lo he hecho nunca.
- Entre 1 y 3 años
- Entre 3 y 6 años
- Entre 6 y 9 años
- Entre 9 y 12 años
- 12 años o más

**¿Qué instrumento o instrumentos tocas?**

- De cuerda frotada (violín,…..)
- De cuerda pulsada (guitarra,…..)
- De viento madera
- De viento metal
- De percusión
- El piano u otro instrumento de teclado
- Canto de forma profesional
- Instrumentos eléctricos o electrónicos (sintetizadores,…)

**Questions included in the study (English version)**

**… How much it helped or prevented you from achieving each one of the goals:**

**The goal: Release and venting of negative emotions (e.g. stress, anxiety, anger)**

Music (e.g. listening, playing an instrument, singing)

| Significantly prevented lo | Prevented | Slightly prevented | Irrelevant | Slightly helped | Helped | Significantly helped |
| --- | --- | --- | --- | --- | --- | --- |
| -3 | -2 | -1 | 0 | 1 | 2 | 3 |

**The goal: Diversion from the crisis**

Music (e.g. listening, playing an instrument, singing)

| Significantly prevented lo | Prevented | Slightly prevented | Irrelevant | Slightly helped | Helped | Significantly helped |
| --- | --- | --- | --- | --- | --- | --- |
| -3 | -2 | -1 | 0 | 1 | 2 | 3 |

**The goal: Enjoyment and maintaining good mood**

Music (e.g. listening, playing an instrument, singing)

| Significantly prevented lo | Prevented | Slightly prevented | Irrelevant | Slightly helped | Helped | Significantly helped |
| --- | --- | --- | --- | --- | --- | --- |
| -3 | -2 | -1 | 0 | 1 | 2 | 3 |

**The goal: Reducing loneliness and creating a sense of "togetherness"**

Music (e.g. listening, playing an instrument, singing)

| Significantly prevented lo | Prevented | Slightly prevented | Irrelevant | Slightly helped | Helped | Significantly helped |
| --- | --- | --- | --- | --- | --- | --- |
| -3 | -2 | -1 | 0 | 1 | 2 | 3 |

**How much is music important to you in general?**

- Not at all important
- Slightly important
- Moderately important
- Very important
- Extremely important

**How much time did you spend on listening to music during lockdown as compared to the time before the crisis?**

- Much less
- Less
- No difference
- More
- Much more

**What is your age?**

- 18 - 24
- 25 - 34
- 35 - 44
- 45 - 54
- 55 - 64
- 65 - 74
- 75 - 84
- +85

**Gender?**

- Male
- Female
- Prefer not to say
- Prefer to self-describe

**With whom did you stay during the lockdown?**

- By myself
- With my partner
- With my core family (children & one or two adults)
- With an extended family (multiple generations or households)
- Friends I don't usually live with
- Flatmates
- I didn't experience a lockdown

**Did you have caring responsibilities?**

- No
- Yes, sole carer for children
- Yes, joint carer for children
- Yes, sole carer for relative/friend
- Yes, joint carer for relative/friend

**Where have you spent the lockdown?**

- Community of Madrid
- Catalonia
- Castilla y León Community
- Castilla la Mancha Community
- Basque Country
- Valencian Community
- Other Communities in Spain
- Abroad. Write where

**Do you consider yourself in a risk group with regard to the  COVID-19 virus?**

- Not at all
- To a very small degree
- To a small degree
- To some degree
- To a large degree
- To a very large degree

**Do you have experience in playing a musical instrument or singing?
If so, how many years have you engaged in lessons or regular practice?**

- No experience
- Between 1 to 3 years
- Between 3 to 6 years
- Between 6 to 9 years
- Between 9 to 12 years
- 12 Years and more

**On which instrument/s?**

- Bow instruments (violin, cello, etc)
- Plucked instruments (Guitar, harp, etc)
- Woodwind instruments
- Brass instruments
- Percussion instruments
- Piano or keyboard
- Professional singing
- Electronic instruments/music technology
